# Secure hemostasis in transhiatal esophagectomy for esophageal cancer with gauze packing

**DOI:** 10.1186/1477-7819-10-276

**Published:** 2012-12-19

**Authors:** Noriyuki Hirahara, Takeshi Matsubara, Yoko Hari, Yusuke Fujii, Hitomi Wake, Yoshitsugu Tajima

**Affiliations:** 1 Department of Digestive and General Surgery, Shimane University Faculty of Medicine, 89-1 Enya-cho, Izumo, Shimane, 693-8501, Japan

**Keywords:** Transhiatal esophagectomy, Hemostasis, Gauze packing

## Abstract

**Background:**

Transhiatal esophagectomy for esophageal cancer implies blind manipulation of the intrathoracic esophagus. We report a secure hemostatic method with gauze packing in transhiatal esophagectomy.

**Methods:**

The gauze-packing technique is utilized for hemostasis just after removal of the thoracic esophagus during transhiatal esophagectomy. After confirming cancer-free margins, the abdominal esophagus and cervical esophagus are transected. A vein stripper is inserted into the oral-side stump of the esophagus and led to exit from the abdominal-side stump of the esophagus. The vein stripper and the oral stump of the esophagus are affixed by silk thread. A polyester tape is then affixed to the vein stripper, as the polyester tape is left in the posterior mediastinum after removal of the esophagus toward the abdominal side. The polyester tape on the cervical side is ligated with gauze and the polyester tape is removed toward the abdominal side. The oral stump of gauze and new additional gauze are affixed. As the first gauze is pulled out from the abdominal side, the second gauze gets drawn from the cervical wound into the mediastinum. The posterior mediastinum is finally packed with gauze and possible bleeding at this site undergoes a complete astriction. The status of hemostasis with the gauze packing is checked by an observation of color and bloodstain on the gauze.

**Results:**

Between January 2005 and February 2012, 13 consecutive patients with esophageal cancer underwent a transhiatal esophagectomy with the gauze-packing hemostatic technique. Hemostasis at the posterior mediastinum was performed successfully and quickly in all cases with this method, requiring up to four pieces of gauze for a complete hemostasis. Median required time for hemostasis was 1219 (range 1896 to 1293) seconds and estimated blood loss was 20.4 (range 15 to 25) ml during gauze packing.

**Conclusions:**

Our technique could minimize bleeding after the removal of the thoracic esophagus. The gauze-packing method is a simple and easy technique for secure hemostasis when performing a transhiatal esophagectomy.

## Background

Transhiatal esophagectomy for esophageal cancer is usually indicated for mucosal cancer with no lymph node metastasis that deviates from an absolute indication for endoscopic treatment. With the recent advances in endoscopic techniques, those indications for esophageal cancer have been extended to surface-layer-spreading lesions and multiple lesions in which endoscopic mucosal or submucosal resection had never been indicated. Therefore, patients eligible for transhiatal esophagectomy are limited [1-4]. Meanwhile, the number of patients with the underlying diseases is rising steadily with the increase in the number of older people. More frequently than before, we have a chance to encounter patients with esophageal cancer in whom differential lung ventilation is infeasible or patients with difficulty receiving open-chest or thoracoscopic surgeries due to anamnesis of thoracoplasty and severe pleurodesis. These patients may be the most suitable candidates for transhiatal esophagectomy as a reductive operation. For these reasons, esophageal surgeons are needed to master this operative procedure. When performing a transhiatal esophagectomy, hemostatic operation is required because this surgical procedure includes blind manipulation of the intrathoracic esophagus. We herein describe a secure hemostatic technique for transhiatal esophagectomy.

## Methods

### Indication

Transhiatal esophagectomy is indicated as a radical surgery for patients with surface-layer-spreading mucosal cancer or multiple early cancer of the esophagus with difficulties receiving endoscopic treatment. Patients for whom the omission of mediastinal lymph node dissection is feasible are also indicated for transhiatal esophagectomy. As a palliative operation, the surgical procedure is used for patients for whom differential lung ventilation is unfeasible due to poor pulmonary function and either open-chest or thoracoscopic surgery is difficult due to such reasons as severe pleurodesis, aged more than 80 years old, and poor performance status.

### Surgical procedure

**Figure 1:**
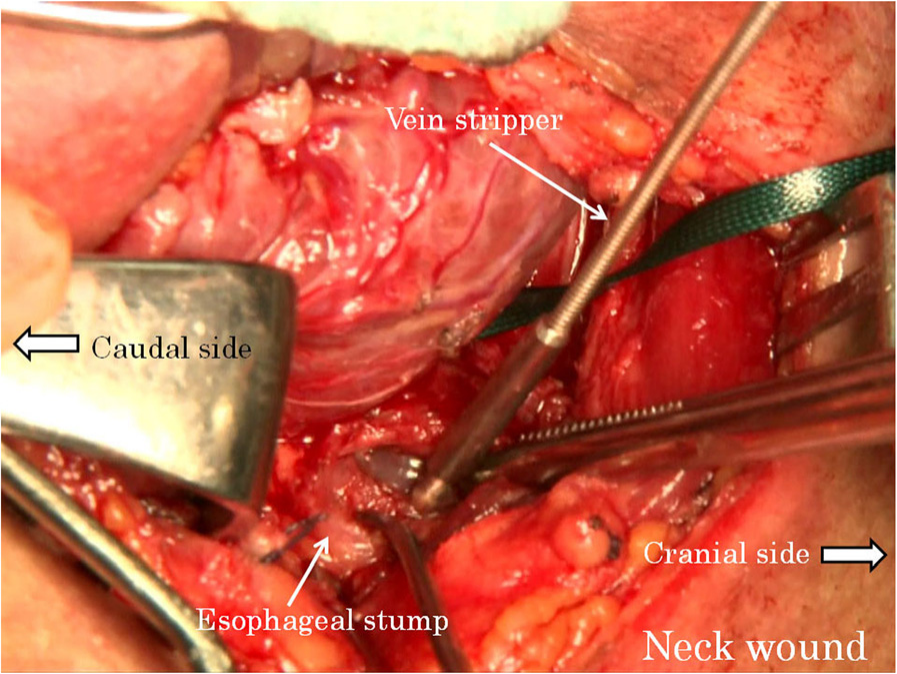
A vein stripper is inserted toward the abdomen from the oral-side stump of the esophagus.

**Figure 2:**
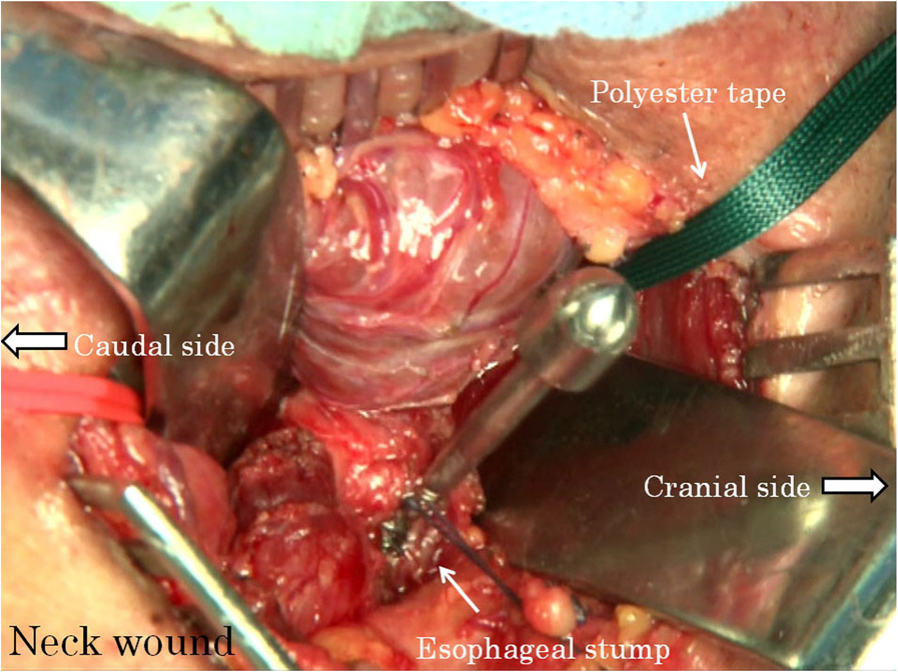
The vein stripper and the oral stump of the esophagus are affixed securely by using silk thread.

**Figure 3:**
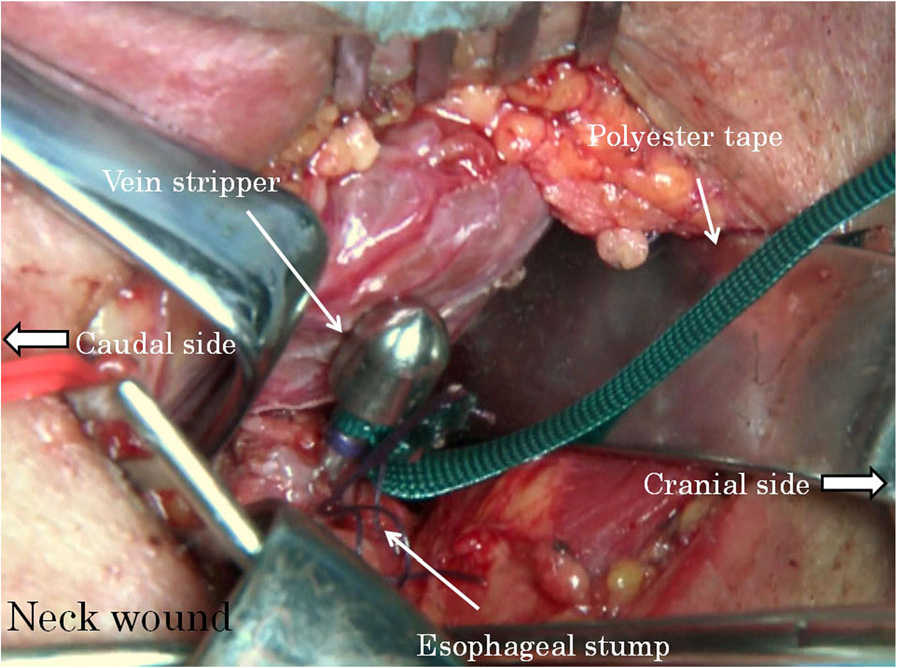
A polyester tape is securely affixed to the vein stripper.

**Figure 4:**
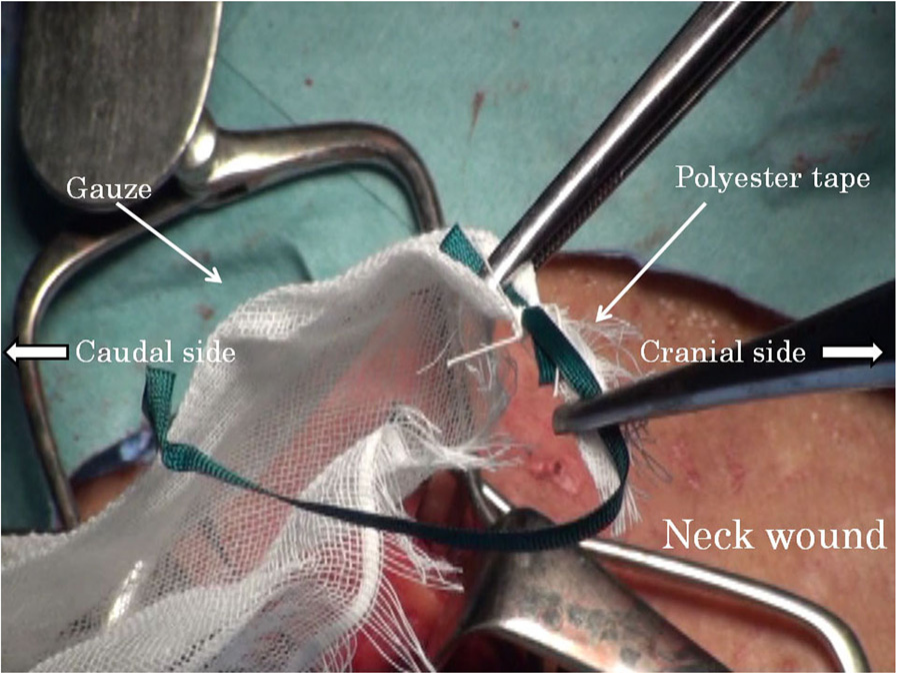
The polyester tape on the cervical side is ligated with gauze.

**Figure 5:**
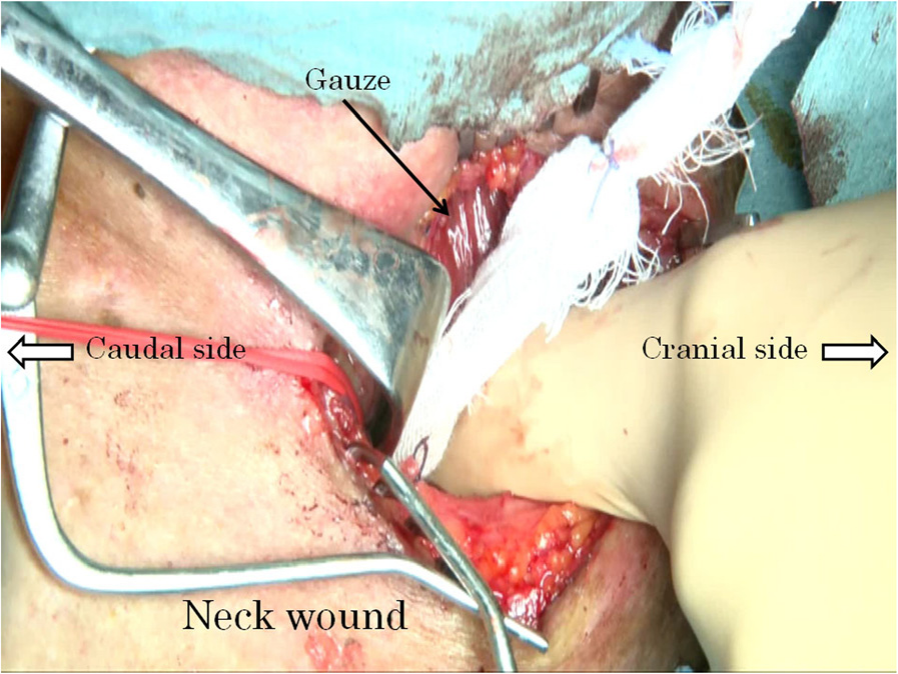
New fixed gauze gets drawn from the cervical wound into the mediastinum.

**Figure 6:**
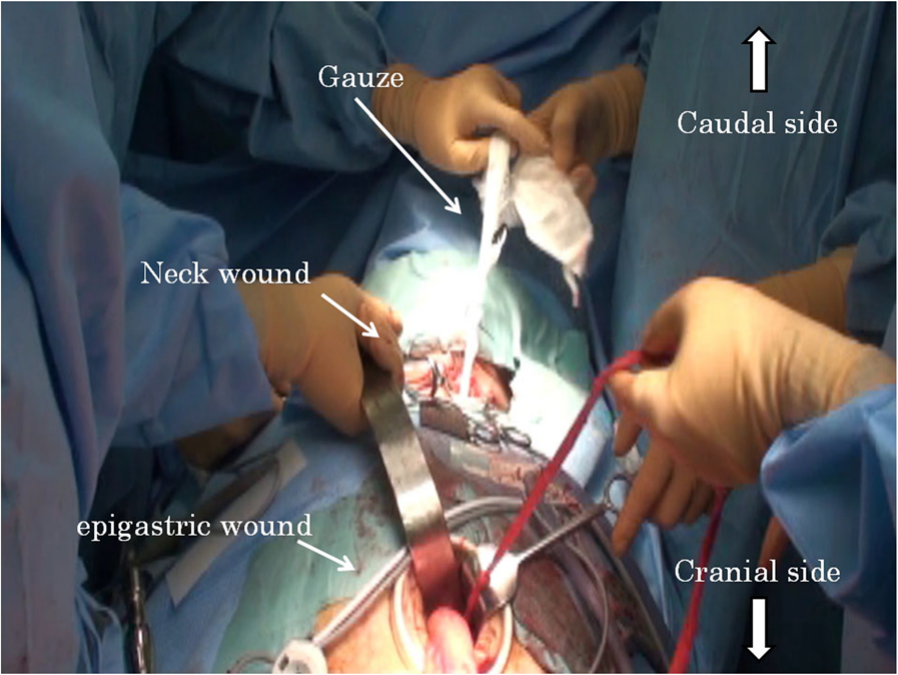
The gauze sufficiently packs the region from which the esophagus has been dissected.

Abdominal manipulation is performed under an upper median incision or laparoscope. The neck manipulation is performed with a collar incision along the skin crease. The lower thoracic esophagus is detached and inferior mediastinal lymph nodes are dissected to the extent possible via the esophageal hiatus with abdominal manipulation. Similarly, the cervical and upper thoracic esophagus is detached and superior mediastinal lymph nodes are dissected with neck manipulation. After confirming the surgical margins free of tumor, abdominal and cervical esophagi are transected, respectively. A vein stripper is inserted toward the abdomen from the oral-side stump of the esophagus and led to exit from the abdominal-side esophageal stump (Figure 1). The vein stripper and the oral stump of the esophagus are affixed securely by using silk thread (Figure 2). A polyester tape longer than the thoracic esophagus is securely affixed to the vein stripper (Figure 3). The thoracic esophagus is then removed toward the abdominal side while confirming that the esophagus can turn. After the thoracic esophagus is removed toward the abdominal side, the polyester tape remains in the posterior mediastinum. Successively, the polyester tape on the cervical side is ligated with gauze and the polyester tape is pulled out toward the abdominal side (Figure 4). The dead space, that is, the posterior mediastinum from which the thoracic esophagus had been pulled out, is sufficiently packed with gauze and bleeding at this site undergoes astriction. About five minutes after the astriction with gauze, the gauze used for astriction and new additional gauze are affixed using a silk thread. As the first gauze is pulled out from the abdominal side, the second gauze gets drawn from the cervical wound into the mediastinum (Figure 5) to pack the posterior mediastinum sufficiently (Figure 6). This procedure is repeated and the status of hemostasis with the gauze packing is checked by an observation of color and bloodstain on the gauze.

Finally, reconstruction of the digestive system is performed and the surgery is completed.

## Results

Between January 2005 and February 2012, 13 consecutive patients with esophageal cancer underwent a transhiatal esophagectomy with the gauze-packing hemostatic technique. Hemostasis at the posterior mediastinum was performed successfully and quickly in all cases with this method, requiring up to four pieces of gauze for a complete hemostasis. Median required time for hemostasis was 1219 (range 1896 to 1293) seconds and estimated blood loss was 20.4 (range 15 to 25) ml during gauze packing. All patients were then reconstructed with a gastric conduit via the posterior mediastinal pathway. None of the patients experienced postoperative rebleeding. The postoperative CT scans showed no effusion in the esophagus-removal region or accumulation of blood in any of the patients.

Between January 2000 and January 2005, 11 consecutive patients with esophageal cancer underwent surgery with conventional hemostatic technique. The drain that was inserted in the posterior mediastinum was slightly bloody for the first two postoperative days, and the quantity of the posterior mediastinum drainage was not less than 100 ml until the eighth postoperative day. However, the drain of all the patients who underwent surgery with the gauze-packing hemostatic technique became serous the day after surgery. The quantity of drainage was less than 100 ml within the fourth postoperative day, and all drains were removed from the patients within the sixth postoperative day.

## Discussion

Turner first reported transhiatal esophagectomy in 1933 as a procedure for dissecting the thoracic esophagus with cervical and abdominal manipulation [5]. Since then, this operative procedure has been applied for many clinical cases of esophagogastric junction cancer in Europe and America and also used as the multidisciplinary therapy with preoperative chemoradiotherapy. A randomized comparative study conducted by Hulscher *et al*. [6] in 2002 reported that transhiatal esophagectomy induced postoperative complications less frequently than the open-chest procedure with mediastinal lymph node dissection. Meanwhile, the prognostic result of transhiatal esophagectomy was shown to be inferior to open-chest surgery, though the difference is not significant [7]. Recently, indications for transhiatal esophagectomy are limited due to the spread of both chemoradiotherapy and endoscopic submucosal dissection. Therefore, the problem with this operative procedure may be operative indications and improvements of long-term prognosis [8-10].

Another problem of transhiatal esophagectomy is accidental bleeding from the proper esophageal artery or from the posterior mediastinum, detached from the esophagus due to the blind manipulation of the intrathoracic esophagus. Because unexpected and uncontrollable bleeding could result in life-threatening complications, an accurate hemostasis is needed in transhiatal esophagectomy. In recent practices, mediastinoscopy and laparoscopy with energy devices are utilized for transhiatal esophagectomy and reconstruction of the digestive system is performed after a confirmation of steady hemostasis [11]. However, the use of energy devices without a clear view entails a risk of secondary damage and an inadvertent use of these items is rather dangerous [12]. The basic hemostatic procedures in surgery are ligation and thermocoagulation of the bleeding points. In cases with unidentifiable bleeding points, such as an extensive oozing, the use of gauze to press the bleeding field is effective [13]. Gauze astriction is a basic hemostatic practice and highly effective, as shown by the common application of gauze packing for emergency operations and disseminated intravascular coagulation associated with massive bleeding. In our method, gauze can be adequately packed into the posterior mediastinum to stop bleeding by pressing the bleeding points. The connective fixation of an additional gauze allows a new piece of gauze to be inserted when necessary, facilitating not only hemostasis but also the assessment of hemostasis. When the posterior mediastinum is selected for the reconstruction route, the detached surface of the esophagus is barely packed by the reconstructive organ and hemostatic effect can be expected. However, astriction effect by the reconstructive organ is not expected when the retrosternal or presternal route is used for reconstruction. The reconstruction routes other than posterior mediastinum thus require a more certain hemostasis. In such situations, our hemostatic method is feasible.

Meanwhile, the insertion of gauze into a limited space could cause secondary damage. However, the azygos vein and bronchial artery do not come in contact with the inserted gauze because they are located across the pleura. Unless a piece of gauze gets forcibly drawn in, the likelihood of damaging other organs is very low.

Up to the present, the gauze-packing technique has been applied in 13 patients. Hemostasis was obtained quickly in all patients. None of the patients experienced postoperative rebleeding. The postoperative CT scan showed no effusion or accumulation of blood in the posterior mediastinum in any of the patients.

The merit of this method compared to conventional hemostatic technique is secure astriction in the middle of the mediastinum where it is distant both from the neck and epigastric wound. The gauze-packing method is a simple and easy technique for secure hemostasis when performing a transhiatal esophagectomy. Our technique could minimize bleeding after the removal of the thoracic esophagus without any accompanying traumatic complications, and it is useful irrespective of the routes and the organs of choice for reconstruction after transhiatal esophagectomy.

## Conclusion

The gauze-packing method is a simple and easy technique for secure hemostasis when performing a transhiatal esophagectomy.

## Consent

Written informed consent was obtained from the patients for publication of this case report and any accompanying images. A copy of the written consent is available for review by the Editor-in-Chief of this journal.

## Competing interests

The authors declare that they have no competing interests.

## Authors’ contributions

NH was the lead author and surgeon for all of the patients. TM and YH gathered information and contributed to the writing of the paper. YF and HW contributed patients and information on the patients. TM and YH were the co-surgeons on the cases. YT reviewed the paper and the surgical technique. All authors read and approved the final manuscript.
